# Six Exonic Variants in the *SLC5A2* Gene Cause Exon Skipping in a Minigene Assay

**DOI:** 10.3389/fgene.2020.585064

**Published:** 2020-11-05

**Authors:** Sai Wang, Yixiu Wang, Jinchao Wang, Zhiying Liu, Ruixiao Zhang, Xiaomeng Shi, Yue Han, Wencong Guo, Irene Bottillo, Leping Shao

**Affiliations:** ^1^Department of Nephrology, The Affiliated Qingdao Municipal Hospital of Qingdao University, Qingdao, China; ^2^Department of Dermatology, Peking University First Hospital, Beijing, China; ^3^Department of Hepatic Surgery, Shanghai Cancer Center, Shanghai Medical College, Fudan University, Shanghai, China; ^4^Yantai Branch of Wenden Osteopathic Hospital, Yantai, China; ^5^Division of Medical Genetics, Department of Molecular Medicine, Sapienza University, San Camillo-Forlanini Hospital, Italy

**Keywords:** *SLC5A2* gene, exonic variant, missense variant, pre-mRNA splicing, minigene analysis, exon skipping

## Abstract

**Background:**

Familial renal glucosuria is a rare renal tubular disorder caused by *SLC5A2* gene variants. Most of them are exonic variants and have been classified as missense variants. However, there is growing evidence that some of these variants can be detrimental by affecting the pre-mRNA splicing process. Therefore, we hypothesize that a certain proportion of *SLC5A2* exonic variants can result in disease *via* interfering with the normal splicing process of the pre-mRNA.

**Methods:**

We used bioinformatics programs to analyze 77 previously described presumed *SLC5A2* missense variants and identified candidate variants that may alter the splicing of pre-mRNA through minigene assays.

**Results:**

Our study indicated six of 7 candidate variants induced splicing alterations. Variants c.216C > A, c.294C > A, c.886G > C, c.932A > G and c.962A > G may disrupt splicing enhancer motifs and generate splicing silencer sequences resulting in the skipping of exon 3. Variants c.305C > T and c.1129G > A probably disturb splice sites leading to exon skipping.

**Conclusion:**

To our knowledge, we report, for the first time, *SLC5A2* exonic variants that produce alterations in pre-mRNA. Our research reinforces the importance of assessing the consequences for putative point variants at the mRNA level. Additionally, we propose that minigenes function analysis may be valuable to evaluate the impact of *SLC5A2* exonic variants on pre-mRNA splicing without patients’ RNA samples.

## Introduction

Familial renal glucosuria (FRG) is a rare renal tubular disease, which is characterized by persistent glucosuria without aberrant glucose metabolism and any other symptoms of tubular malfunction (Calado et al., 2008; Aires et al., 2015; Wang et al., 2019). The vast majority of FRG patients are associated with *SLC5A2* (OMIM 182381) pathogenic variants (Calado et al., 2008; Sada et al., 2019; Wang et al., 2019). The full-length *SLC5A2* gene is 7.7 kb located on chromosome 16p11.2 and encodes for a 672 amino acid low-affinity sodium/glucose co-transporter 2 (SGLT2) with a total of 14 exons (Wells et al., 1993). SGLT2 is mainly expressed in the brush border of renal proximal tubules, couples with Na^+^ and glucose at a ratio of 1:1 (Yu et al., 2020), and reabsorbs most of filtered renal glucose (van den Heuvel et al., 2002).

Among 83 *SLC5A2* different variants described in the Human Gene Mutation Database (HGMD, accessed April 2019), 59 are missense/non-sense variants accounting for 77% (64/83). The remaining variants are splicing (5, 6%), small deletions (7, 8%), small insertions (3, 4%), small indels (1, 1%) and gross deletions (3, 4%). Most mutation analyses are performed mainly at genome level, and the impact of a variant on the encoded mRNA and protein is only predicted from the DNA sequence (López-Bigas et al., 2005). Only in few cases have the effects of variants been experimentally confirmed at both DNA and RNA levels.

Generally, exonic point variants are classified as missense, synonymous, silent, or non-sense variants, and certain point variants cause abnormal precursor-mRNA (pre-mRNA) splicing, a key step in gene expression, and this has been associated to the pathogenesis of various disorders (Takeuchi et al., 2015; Gonzalez-Paredes et al., 2016; Shao et al., 2018; Han et al., 2019; Wang et al., 2020). The pre-mRNA splicing process can be changed by point variants, which disrupt canonical splice sites (5′ donor site, 3′ acceptor site and branch site) and polypyrimidine tract (Gonzalez-Paredes et al., 2014, 2016); or creating or deactivating sequences that regulate splicing, such as exonic splicing enhancers/silencers (ESEs/ESSs) or intronic splicing enhancers/silencers (ISEs/ISSs) (Cartegni et al., 2002; Gonzalez-Paredes et al., 2014; Takeuchi et al., 2015). Furthermore, the substitution of some nucleotides in intronic and exonic regions can create or activate new cryptic splice sites, which may alter the final configuration of the mRNA (Gaildrat et al., 2010).

The ideal experimental method to identify splicing alterations is to analyze RNA from patients. However, in many cases, this type of sample is not always available from the patient or it has been obtained in ways that cannot ensure its stability. Alternatively, minigene analysis has become an approach to initially assess whether a particular variant affects pre-mRNA splicing. In a previous study, we have used this method to assess the consequences of presumed missense *SLC12A1* variants on splicing and confirmed that one missense variant actually caused abnormal splicing (Han et al., 2019).

Since a large number of *SLC5A2* exonic variants described lack studies on the effects of pre-mRNA, we hypothesized that some *SLC5A2* variants that have been reported as missense or synonymous can change splicing process through modification of splice sites or splicing regulatory sequences present in the pre-mRNA molecules. This study may provide new insights to the functional consequences of previously described *SLC5A2* exonic point variants on pre-mRNA splicing with bioinformatics tools and minigene assays.

## Materials and Methods

The nomenclature of variants followed the guidelines of the Human Genome Variation Society^1^. The number of nucleotides was based on the *SLC5A2* cDNA sequence (GenBank accession number NM_003041.4), with c.1 representing the first position of the translation initiation codon.

### *In silico* Prediction and Screening Criteria

All *SLC5A2* missense variants were selected from the Human Gene Mutation Database (March 2019) and literature (Dhayat et al., 2016; Yu et al., 2016a,b; Gong et al., 2017; Wang et al., 2017), except c.331T > C p.(Trp111Arg), c.374T > C p.(Met125Thr), c.393G > C p.(Lys131Asn), c.394C > T p.(Arg132Cys), c.612G > C p.(Gln204His), c.829C > T p.(Pro277Ser), c.880G > A p.(Asp294Asn), c.1003A > G p.(Ser335Gly), c.1129G > A p.(Gly377Ser), c.1194C > A p.(Phe398Leu), c.1343A > G p.(Gln448Arg), c.1573C > T p.(His525Tyr) and c.1739G > A p.(Gly580Asp), which were identified in our patients (Zhao et al., 2016; Wang et al., 2019). These variants were analyzed through online bioinformatics software to determine their possible effects on pre-mRNA processing. To analyze the potential effect of a variant on consensus 5′ donor or 3′ acceptor site and to predict the generation and/or activation of novel sites, *in silico* analysis by BDGP^2^ were performed. Human Splicing Finder version 3.1 (HSF)^3^ were used to investigate the possible impact of putative missense alterations on splicing regulatory sequences, such as ESEs and ESSs.

In this study, we selected *SLC5A2* variants for experimental analyses according to the following criteria: (1) close to the 5′ or 3′ ends of exons; (2) predicted effect of the variant on exonic splicing regulatory elements (ESEs broken or new ESSs creation).

### Amplification of *SLC5A2* Genomic Fragments

Genomic DNA was extracted from peripheral blood leukocytes of healthy controls by GenElute blood genomic DNA extraction kit (Sigma, NA2010) according to the manufacturer’s instruction. For the *in vitro* splicing assay, the target exons including approximately 50–200 nucleotides flanking shortened introns were amplified through specific oligonucleotide primers with *Xho*I and *Nhe*I restriction sites (*Xho*I: CCGC^CTCGAG; *Nhe*I: CTAG^CTAGC). Both edges of the shortened introns were properly designed by the HSF program so as to avoid the activation of cryptic splicing. The primers were designed by web-based source Primer-Blast^4^ and were listed in Supplementary Table 1. DNA extraction from the healthy control was performed with complete understanding and written consent of the subject and was approved by the Ethics Committee of the Affiliated Qingdao Municipal Hospital of Qingdao University prior to participation in the study.

### Minigene Constructions and Site-Directed Mutagenesis

PCR fragments were purified with Gel Extraction kit (Cwbio, China). Purified products and pSPL3 exon trapping vector were separately digested by restriction enzymes *Xho*I and *Nhe*I (*Xho*I: CCGC^TCGAG; *Nhe*I: CTAG^CTAGC). Ligation reactions were performed using 0.2 U of T4 DNA ligase (Takara, Japan) with overnight incubation at 16°C. After that, the vector with cloned insert were transformed into DH5α competent E. coli cells and multiplied in the Luria-Bertani broth and spread evenly on the IPTG/x-GAL (Invitrogen, United States) coated ampicillin-Luria-Bertani agar plates for 16-h at 37°C (Zhang et al., 2018). The extraction of the collected monoclonal colonies was performed using PurePlasmid Mini Kit (Cwbio, China). Minigenes were then sequenced using forward and reverse primers. Chromas 2.31 and Vector NTI Advance 10 were used for sequence analysis and alignment.

Variants of interest were introduced into *SLC5A2* exons with QuikChange II Site Directed Mutagenesis Kit (Stratagene, La Jolla, CA, United States) following the manufacture’s recommendations. Mutagenesis primers were designed using Primer X^5^ (Supplementary Table 2). Primer extension and PCR amplification reactions are as follows: the first step is denaturation at 95°C for 30 s, followed by 33 cycles, denaturation at 95°C for 30 s, annealing at 62°C–53°C for 30 s, elongation at 72°C for 7 min, and finally extension at 72°C for 5 min. In order to determine the existence of target variants, all products were confirmed through direct sequencing.

### Minigene Splicing Assay

Human embryonal kidney 293T (HEK293T) and Hela cells were cultured in DMEM with high glucose (4.5 g/L), supplemented with 10% fetal bovine serum and incubated at 37°C in a 5% CO_2_ incubator. One day before transfection, cells were seeded on 24-well plate to grow to 70–80% confluence in an antibiotic-free medium. Each group of minigenes (empty pSPL3 control, wild-type and mutant) were transfected to HEK239T and Hela cells with Lipofectamine 2000 (Invitrogen, United States) following the manufacturer’s instructions. Forty-eight hours after transfection, total RNA was extracted with TRIzol reagent (Invitrogen, United States). First-strand cDNA synthesis was carried out through random-primed reverse transcription using PrimeScript 1st Strand cDNA Synthesis kit (Takara, Japan) (Shao et al., 2018). The resulting cDNA was amplified by PCR using vector-specific primers: SD6 (the forward primer: 5′-TCTGAGTCACCTGGACAACC-3′) and SA2 (the reverse primer: 5′-ATCTCAGTGGTATTTGTGAGC-3′). The PCR amplification reaction was performed as follows: in 50 μL volume, 2 μL of cDNA, 10 μL of 5 × PrimerSTAR Buffer (TaKaRa, Japan), 1 μM of each primer, 0.8 μM dNTPs and 0.5 μL PrimerSTAR HS DNA Polymerase (TaKaRa, Japan) in a 9700 (Applied Biosystem, United States). Thermal conditions were 29 cycles of 98°C for 30 s, 58°C for 30 s, and elongation at 72°C for 90 s, preceded by 30 s at 98°C, and followed by a final elongation step at 72°C for 10 min. PCR products were resolved by electrophoresis through 1.5% agarose gel and each band signal was quantified by the software Quantity One (Bio-Rad, United States). All transcripts were analyzed by DNA sequencing. The bioinformatics online software Basic Local Alignment Search Tool was used to compare DNA sequences with the reference *SLC5A2* sequence (GenBank accession number NM_003041.4). Quantification of the abnormal splicing percentage was densitometrically calculated as the percentage of exclusion (%) = (lower band/[lower band + upper band]) × 100. Error bars represent SEM (*n* = 3). ^∗^*P* < 0.05, unpaired Student’s *t*-test.

## Results

**FIGURE 1:**
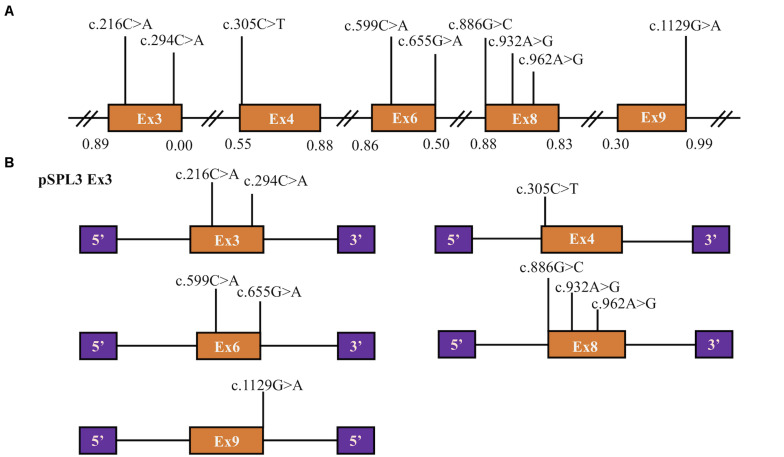
Position of presumed missense variants that we analyzed and the schematic diagram of *SLC5A2* minigenes. **(A)** Orange boxes and black lines between them represent the coding exons and introns sequences, respectively. Their sizes are out of proportion. The BDGP scores of donor and acceptor splice sites are represented in decimal. **(B)** Schematic representation of five minigenes constructed by pSPL3 vector and *SLC5A2* wild-type sequences (orange boxes) including exon 3 (pSPL3 Ex3), exon 4 (pSPL3 Ex4), exon 6 (pSPL3 Ex6), exon 8 (pSPL3 Ex8) and exon 9 (pSPL3 Ex9), respectively. Purple boxes depict 5′ and 3′ exons of pSPL3 vector. Black lines indicate intron sequences.

**FIGURE 2:**
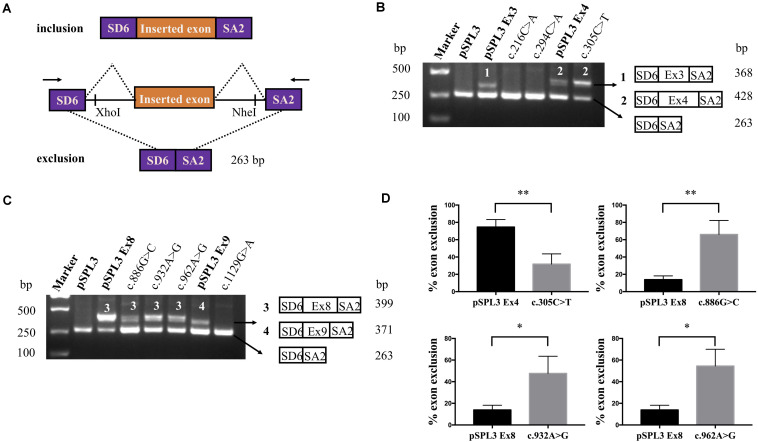
RT-PCR results and DNA sequencing of *SLC5A2* exonic variants. **(A)** RT-PCR amplified products of hybrid minigene transcripts in HEK293T cells. The transcripts produced by the hybrid minigene are schematically shown and the arrows show the primers used to amplify (inset). **(B,C)** Gel electrophoresis of the RT-PCR product of minigene transcripts in HEK293T cells. **(B)** Lane 1: marker; Lane 2: pSPL3 (263 bp); Lane 3: pSPL3 Ex3 (368 bp and 263 bp); Lane 4: c.216C > A (263 bp); Lane 5: c.294C > A (263 bp); Lane 6: pSPL3 Ex4 (428 bp and 263 bp); Lane 7: c.305C > T (428 bp and 263 bp). **(C)** Lane 1: marker; Lane 2: pSPL3 (263 bp); Lane 3: pSPL3 Ex8 (399 bp and 263 bp); Lane 4: c.886G > C (399 bp and 263 bp); Lane 5: c.932A > G (399 bp and 263 bp); Lane 6: c.962A > G (399 bp and 263 bp); Lane 7: pSPL3 Ex9 (371 bp and 263 bp); Lane 8: c.1129G > A (263 bp). All assays were performed in triplicate. **(D)** Quantification of the splicing percentage in the graph was densitometrically calculated on a molar basis as the percentage of exclusion (%) = (lower band/[lower band + upper band]) × 100. Error bars represent SEM (*n* = 3). ^∗^*P* < 0.05, ^∗∗^*P* < 0.01, unpaired Student’s *t*-test.

A total of 77 missense variants compiled in the *SLC5A2* database were analyzed with the bioinformatics software. We eliminated two of these variants [c.1891G > A p.(Glu631Lys) and c.1961A > G p.(Asn654Ser)] since they were located in the last exon and therefore could not be analyzed with the minigene approach. We finally selected the variants within two bases of 5′ or 3′ ends of the exons, or these variants were predicted to have an effect on splicing regulatory elements according to HSF (The total number of disrupted ESEs and gained ESSs is more than 5). Finally, 9 missense variants [c.216C > A p.(Phe72Leu), c.294C > A p.(Phe98Leu), c.305C > T p.(Ala102Val), c.599C > A p.(Thr200Lys), c.655G > A p.(Ala219Thr), c.886G > C p.(Val296Leu), c.932A > G p.(Lys311Arg), c.962A > G p.(Lys321Arg) and c.1129G > A p.(Gly377Ser)] located in five exons of the *SLC5A2* gene were included in this study (Table 1 and Figure 1A). *In silico* prediction, the results of these variants are shown in Table 1. Except for the minigene we have previously constructed [pSPL3 Ex8, intron 7 (207 bp)-exon 8 (136 bp)-intron 8 (186 bp)] (Zhao et al., 2016), four control minigenes containing 363, 537, 379, and 409 bp *SLC5A2* genomic inserts were constructed respectively, with the following structure: (pSPL3 Ex3, intron 2 (55 bp)-exon 3 (105 bp)-intron 3 (203 bp); pSPL3 Ex4, intron 3 (172 bp)-exon 4 (165 bp)-intron 4 (200 bp); pSPL3 Ex6, intron 5 (194 bp)-exon 6 (81 bp)-intron 6 (104 bp); pSPL3 Ex9, intron 8 (190 bp)-exon 9 (108 bp)-intron 9 (111 bp). Using the corresponding wild-type (WT) minigenes as a template, seven mutant minigenes were generated through site-directed mutagenesis (Figure 1B). Regrettably, two mutant minigenes were not introduced into the pSPL3 Ex6 minigene by this approach [c.599C > A p.(Thr200Lys) and c.655G > A p.(Ala219Thr)] due to experimental technique. The results of the RT-PCR experiments indicated that all of them disturbed normal pre-mRNA splicing products *in vitro* (Figures 2B,C). Among seven candidates selected by BDGP and HSF programs *in silico*, six variants [c.216C > A p.(Phe72Leu), c.294C > A p.(Phe98Leu), c.886G > C p.(Val296Leu), c.932A > G p.(Lys311Arg), c.962A > G p.(Lys321Arg) and c.1129G > A p.(Gly377Ser)] caused exon skipping (Figures 2B,C). One variant [c.305C > T p.(Ala102Val)] caused an increase in exon inclusion compared with WT (Figure 2B).

**TABLE 1:** *SLC5A2* exonic variants selected from this study and their effects.

**Variant**	**Exon**	**Exon length (bp)**	**bp from exon end**	**BDGP**		**HSF**		**References**
				**5′ donor site**	**3′ acceptor site**	**Gained ESSs^1^**	**Disrupted ESEs^1^**	
c.216C > A	3	105	+18	0	0.89	3	3	Santer, 2003
c.294C > A	3	105	−10	0	0.89	4	2	Yu et al., 2011
c.305C > T	4	165	+2	0.88	0.55	4	0	Calado et al., 2006
c.599C > A	6	81	+25	0.50	0.86	2	4	Kleta et al., 2004
c.655G > A	6	81	−1	0.50	0.86	0	0	Kleta et al., 2004
c.886G > C	8	136	+1	0.83	0.88	0	0	Rebelo et al., 2012
c.932A > G	8	136	+47	0.83	0.88	2	4	Santer et al., 2003
c.962A > G	8	136	−60	0.83	0.88	3	4	Magen et al., 2005
c.1129G > A	9	108	−1	0.99	0.30	0	0	Wang et al., 2019

*ESE, exonic splicing enhancer; ESS, exonic splicing silencer. Numbers with “+” are distance from the 5′ end of the exon and those with “−” are distance from the 3′ end.^1^Values 0, 2, 3 and 4 indicate number of splicing regulatory elements gained or disrupted.*

### Presumed Missense Variants c.216C > A p.(Phe72Leu) and c.294C > A p.(Phe98Leu) Cause Skipping of Exon 3

Two variants located in exon 3 were included in this study (Table 1 and Figure 1). Variant c.216C > A p.(Phe72Leu) is located at position 18 in exon 3, which contains three possible overlapping ESE sites, TCTTC, CGCCTC and CGCCT (according to HSF, the affected base are underlined). Bioinformatics analysis of this variant indicated the disruption of these ESEs and the generation of three new ESS sites (TCTCTTAG, TCTTAG and TAGCCA), which corresponded to a Sironi’s type 3 inhibitory sequence and a presumed binding site of the splicing repressor heterogeneous nuclear ribonucleoprotein A1 (Table 1). Presumed missense variant c.294C > A p.(Phe98Leu) is located 10 bp upstream from an extremely weak donor site in exon 3 (score: 0.00) (Table 1). The results of HSF analysis indicate that this variant inactivates two potential ESEs site (TTCGAG, CGAGTGG) and generates four potential overlapping ESSs (GATTAG, TTAGAGTG, TAGAGT and TAGAGTG) corresponding to a Sironi’s type 1 inhibitory sequence (TTAGAGTG, 68.40%) and a new hnRNPA1 binding site (TAGAGT, 87.62%) (Table 1). The effect of both variants on pre-mRNA splicing were studied using control (pSPL3 Ex3) and mutant (c.216C > A and c.294C > A) minigenes. RT-PCR analysis of minigenes after transfection indicated the control minigene produced two different bands, whereas both mutant minigenes generated a unique product (Figure 2). Sequencing analysis of all bands showed that the larger fragment of 368 bp was a transcript containing exon 3, while the smaller splice of 263 bp was a transcript without exon 3. The absence of exon 3 in the mutant transcript did not alter the open reading frame. The SGLT2 protein encoded by this altered mRNA would lead to the loss of 35 amino acids (residues 67–101). Therefore, we postulate both variants result in the skipping of exon 3 because of ESEs destruction and ESSs generation.

### Variant c.1129G > A p.(Gly377Ser) Leads to Exon 9 Skipping

Presumed *SLC5A2* missense variant c.1129G > A p.(Gly377Ser) is caused by the last nucleotide substitution in exon 9. Bioinformatic analysis with BDGP demonstrated that this variant marginally reduces the score of the WT 5′ splice site from 0.99 to 0.94 (Table 1). Additionally, analysis of this variant with HSF predicted the alteration of the donor site. Taken together, to examine the experimental effect of variant c.1129G > A, we used a minigene containing exon 9 and surrounding intronic sequences. RT-PCR analysis results showed the splicing products produced by the mutant and WT minigenes were different. The WT lane demonstrated 2 different fragments of 263 and 371 bp, respectively (Figure 2C). Direct sequencing results showed that the larger fragment contained *SLC5A2* exon 9 flanked by two exons of the pSPL3 vector, while the smaller one included only the 3′ and 5′ pSPL3 exons. In contract, the mutant lane revealed one unique fragment of 263 bp corresponding to skipping of exon 9 in the mRNA. The deletion of exon 9 in the mutant transcript would cause an abnormal ligation of exons 8 and 10, resulting in the loss of 36 amino acids (residues 341–376) at the protein level without altering the open reading frame. Therefore, variant c.1129G > A p.(Gly377Ser) abrogates the donor splice site and causes exon 9 skipping.

### Variants c.305C > T p.(Ala102Val), c.886G > C p.(Val296Leu), c.932A > G p.(Lys311Arg) and c.962A > G p.(Lys321Arg) Altered the Amounts of the Exon-Excluded Transcripts Compared With Those of the WT Plasmids

The variant c.305C > T p.(Ala102Val) affected the C nucleotide at position 2 of exon 4. *In silico* prediction showed that this substitution increased the score of the WT 3′ splice site from 0.55 to 0.74. The variant c.886G > C p.(Val296Leu) involved the first nucleotide of *SLC5A2* exon 8, which lied downstream of the conserved AG dinucleotide. Analysis of this variant with BDGP indicated that it abolished the acceptor splice site of intron 7 (mutant score: 0.40 compared to the wild-type score: 0.88). The other two variants, c.932A > G p.(Lys311Arg) and c.962A > G p.(Lys321Arg), located in exon 8 were predicted by HSF to alter ESEs (two and three, respectively) and create four ESSs. In order to verify whether these variants affected mRNA splicing, we also carried out minigene splicing experiments *in vitro*. As a result, two fragments were uniquely detected from the RT-PCR products of the WT and the mutant. Direct sequencing of all products showed that the larger amplicons were the exons-included transcripts and the smaller amplicons are the exons-excluded transcripts (Figures 2B,C). Analysis of cDNA prepared from HEK293T and Hela cells revealed that the amounts of the exon 4-skipping transcript of c.305C > T were significantly decreased with those of the control plasmid (74.5 versus 31.8% in HEK293), whereas there are a significant increase of exon 8-skipping in c.886G > C, c.932A > G and c.962A > G (13.9 versus 66.0%; 13.9 versus 47.7% and 13.9 versus 54.5%, respectively) (Figure 2D). These data strongly suggested that these exonic variants disturbed the normal splicing *in vitro*.

## Discussion

Pre-mRNA splicing is a key process in eukaryotic gene expression, which removes introns and ligates exons successively. This process is promoted by a ribonucleotide complex, called spliceosome, which interacts with specific RNA sequences at exon-intron boundaries to precisely and efficiently control intron deletion and exon inclusion and produces correct mature mRNAs (Baralle and Baralle, 2018). Misrecognition of exon-intron boundaries or failure to eliminate introns generate aberrant mRNAs that either encode faulty protein or are degraded. In mature mRNAs, the exons inclusion depends on intrinsic regulatory sequences. Variants within the cis-motifs can disrupt the splicing process and induce disease phenotypes in human. The important part of splicing variants reflects the necessity to characterize variants at the mRNA level in that exonic variants away from the canonical GT-AG splice site could certainly be misclassified as missense variants if only the DNA is examined. In fact, it has been estimated that approximately 25–50% of exonic mutations cause disease by affecting normal pre-mRNA splicing (López-Bigas et al., 2005; Brierley and Steensma, 2016).

The purpose of this research was to evaluate the effect of *SLC5A2* exonic variants associated with FRG on the splicing process with minigene systems and bioinformatics tools. We assumed that some *SLC5A2* variants initially described as missense alterations could also affect pre-mRNA splicing. As far as we know, in the *SLC5A2* gene, no such research had been reported. Therefore, we constructed pSPL3 minigene reporter vector to determine whether an exonic variant affects splicing efficiency. The minigene, including a conventional expression system with two cassette exons (SD6 and SA2), is used to analyze the resultant mRNA transcripts. It mainly generates two transcripts. One is composed of exon SD6, an inserted exon and exon SA2 (upper), and the other is composed only of exon SD6 and SA2 (lower) (Figure 2A). After the minigenes inserted with targeted variants were transfected to HEK293T and Hela cells, total RNA was extracted and transcribed to cDNA. As a result, all missense variants studied changed normal splicing, and 6 of them cause exon skipping. However, we should keep it in mind that the minigene strategy has a methodological limitation and could not detect all splicing patterns as a result of this, although it is an efficient tool for the detection of splicing defects.

Variants c.216C > A p.(Phe72Leu) and c.294C > A p.(Phe98Leu) were previously identified as missense variants p.(Phe72Leu) and p.(Phe98Leu), respectively (Santer, 2003; Yu et al., 2011). The variant p.(Phe72Leu) affects highly conserved amino acid residue in the transmembrane helices (TMHs) 2 of SGLT2, while p.(Phe98Leu) is located in the extracellular loop (between TMH 2 and TMH 3). Both variants were found to influence related ESEs and ESSs motifs by the assessment of HSF. The results of our minigenes indicated that both variants produced the same transcript lacking the entire exon 3. As has been shown in other cases, many juxtaposed regulatory sequences including ESEs and ESSs regulate exon usage in a combinatorial manner. They promote or inhibit the identification of surrounding splice sites through recruiting diverse protein factors (Shao et al., 2018). In this study, we suspect that these exonic base substitutions may destroy a variety of ESEs and generate multiple ESSs, causing a significant reduction in the proportion of ESEs/ESSs. Consequently, the total strength of identifying and using adjacent splice site is prominently decreased. In addition, exon 3 had a weak 5′ splice donor site (score 0.00, assessed by BDGP, Table 1 and Figure 1). In the context of the weak splicing site, the exon-intron boundary of exon 3 may be not correctly recognized without the need for any assistance of the ESEs. The effect on the SGLT2 protein of joining exons 2 and 4 would lose 35 amino acids and does not alter the open reading frame. Therefore, these mutant proteins would lack part of TMH2 and part of the extracellular loop. Of note, *Yu et al*. reported that *SLC5A2* alternative transcript lacking exon 3 identified in human cells diminishes expression of in the apical membrane of proximal tubules of kidney (Yu et al., 2011). Consequently, we consider that variants c.216C > A and c.294C > A cause disease due to the aberrant splicing.

Variant c.1129G > A was identified by us and categorized as missense variant p.(Gly377Ser), which influenced the last nucleotide of exon 9. Such substitutions often have an adverse effect on the recognition of canonical splice sites by the cell mechanism (Gonzalez-Paredes et al., 2016). Bioinformatics analyses indicated that the authentic donor splice of intron 9 may be affected. Furthermore, we demonstrated that this exonic variant disturbed the normal splicing *in vitro* causing exon 9 skipping. Subsequently, the ligation of exons 8 and 10 would result in a lack of 36 amino acids and the production of a truncated protein. As a result, this mutated SGLT2 protein would lack the part of the extracellular loop and may reduce or abolish the transport activity of SGLT2.

Variant c.305C > T p.(Ala102Val) disturbed normal splicing in the minigene assay, resulting in an increase of the exon 4-included transcript, which may be due to the enhanced recognition of the 3′ splice site of intron 3. Predicting the impact of this splicing modification is difficult, however, in other genes, a small number of exon inclusions induced by variants have also been described, some of which may cause significant clinical symptoms (Vezain et al., 2010; Soukarieh et al., 2016; Perdomo-Ramirez et al., 2019). The minigene assays showed that variants c.886G > C p.(Val296Leu), c.932A > G p.(Lys311Arg) and c.962A > G p.(Lys321Arg) altered normal splicing by increasing approximately 52, 34, and 41% exon 8 exclusion compared with WT, respectively. We hypothesized that the reason for the exon 8 skipping caused by variant c.886G > C probably was that it abolished the acceptor splice site of intron 7. In addition, we also speculate the disruption of functional ESEs and/or the generation of functional ESSs may be the reason for the aberrant transcripts of variants c.932A > G and c.962A > G that lack exon 8. The complete skipping of exon 8 results in a 45 amino acid deletion (residues 296–340) with a subsequent frameshift from codon 341 and premature termination at position 371 in exon 9. Therefore, these variants probably have a double destructive effect; The deletion of exon 8 would result in a truncated protein lacking the COOH-terminal domain from TMH 8 to TMH 14 in the mutant SGLT2 protein and the remaining mRNA is damaged due to the resulting amino acid alterations.

In this study, 7 of the nine *SLC5A2* variants were tested *via* minigene assays. From the results, the software HSF 3.1 seems to be suitable to predict the effect of *SLC5A2* exonic mutations on pre-mRNA splicing. Although the results of several studies have showed almost 100% concordance between the results obtained with the analysis of patients’ RNA and those from cells transfected with minigenes (Steffensen et al., 2014; van der Klift et al., 2015; Nakanishi et al., 2017; Perdomo-Ramirez et al., 2019), the best approach to determine whether a nucleotide substitution or allelic variant affects splicing is to assay splicing of the endogenous RNA from the relevant tissue of affected individuals. In addition, the shortcomings of this study are that we did not introduce these variants in cDNA according to prediction of single amino acid alterations and the exon exclusion and tested for SGLT2 glucose transport activity and cell surface expression. Further investigation is needed to determine the functional activities of these mutant SGLT2.

In conclusion, our results revealed that 7 previously presumed missense *SLC5A2* variants altered pre-mRNA splicing with bioinformatics tools and minigenes. Variants c.216C > A, c.294C > A, c.886G > C, c.932A > G and c.962A > G may disrupt splicing enhancer motifs and generate splicing silencer sequences resulting in skipping of exon 3. Variants c.305C > T and c.1129G > A probably disturb a 3′ acceptor and a 5′ donor splice site leading to exon skipping, respectively. To our knowledge, we report, for the first time, *SLC5A2* exonic variants affecting pre-mRNA splicing and we propose that these variants should be categorized as splicing variants. Furthermore, these findings emphasize the necessity of evaluating the impact of missense variants on mRNA in FRG, and without patients’ RNA samples, a minigene assay may be a valuable tool for assessing the impact of *SLC5A2* exonic variants on pre-mRNA splicing.

## Data Availability Statement

The datasets presented in this study can be found in online repositories. The names of the repository/repositories and accession number(s) can be found in the article/Supplementary Material.

## Ethics Statement

The studies involving human participants were reviewed and approved by Affiliated Qingdao Municipal Hospital of Qingdao University. The participants provided their written informed consent to participate in this study.

## Author Contributions

SW conceived and designed the experiments and wrote the manuscript. YW and JW performed the experiments. ZL, RZ, XS, YH, IB, and WG were involved in the data analysis. LS revised the manuscript. All authors had read and approved the final manuscript.

## Conflict of Interest

The authors declare that the research was conducted in the absence of any commercial or financial relationships that could be construed as a potential conflict of interest.

## Footnotes

1 http://varnomen.hgvs.org

2 http://www.fruitfly.org

3 http://www.umd.be/HSF/

4 http://www.ncbi.nlm.nih.gov/tools/primer-blast

5 http://www.bioinformatics.org
