# Supplementary material for: Six Exonic Variants in the SLC5A2 Gene Cause Exon Skipping in a Minigene Assay
Source: Front Genet. 2020 Nov 5;11:585064. doi: 10.3389/fgene.2020.585064 (PMC7674938; doi:10.3389/fgene.2020.585064)
Supplement: Supplementary file 1 [file Data_Sheet_1.docx]

**Supplementary Material**

**Supplementary Table 1.** Primer sequences used to amplify *SLC5A2* genomic fragments

| Location | Forward primer sequence | Reverse primer sequence | Product size (bp) |
| --- | --- | --- | --- |
| Exon 3 | 5’-CCGC^TCGAGGAACGGGAGGGGCCT-3’ | 5’-CTAG^CTAGCCAGAAGCAGGACCAACAGT-3’ | 364 |
| Exon 4 | 5’-CCGC^TCGAGCAGCTCTGTTCCTTGGTGC-3’ | 5’-CTAG^CTAGCCTAGCCTCAAAGCTTCCCTC-3’ | 537 |
| Exon 6 | 5’-CCGC^TCGAGAAACTCAGCCACACTCTGC-3’ | 5’-CTAG^CTAGCCCTCCTTAGGACCCTCAGAC-3’ | 379 |
| Exon 8 | 5’-CCGC^TCGACGCCTTCCCCACAACGGTC-3’ | 5’-CTAG^CTAGCCGTTAGGACGGGGCCTGG-3’ | 529 |
| Exon 9 | 5’-CCGC^TCGAGCCTTTCCTGTGCCAGCAAC-3’ | 5’-CTAGC^TAGCTGAGGCTGGGGCCACT-3’ | 409 |

**Supplementary Table 2.** Primer sequences of site-directed mutagenesis

| Mutation | Location | Forward primer sequence | Reverse primer sequence |
| --- | --- | --- | --- |
| c.216C>A | Exon 3 | 5’-GCCTCTCTCTTAGCCAGCAACATC-3’ | 5’-GATGTTGCTGGCTAAGAGAGAGGC-3’ |
| c.294C>A | Exon 3 | 5’-CTGTTGCTGGATTAGAGTGGAATGTGAG-3’ | 5’-CTCACATTCCACTCTAATCCAGCAACAG-3’ |
| c.305C>T | Exon 4 | 5’-CCCGTAGGTGCTCTTCGT-3’ | 5’-ACGAAGAGCACCTACGGG-3’ |
| c.599C>A | Exon 6 | 5’-CGCTGATGTACAAGGACACGGTACA-3’ | 5’-TGTACCGTGTCCTTGTACATCAGCG-3’ |
| c.655G>A | Exon 6 | 5’-CATGGGTTACAGTAGGGGCTCG-3’ | 5’-CGAGCCCCTACTGTAACCCATG-3’ |
| c.886G>C | Exon 8 | 5’-CCCTCCCGTAGCTCATCGTGCAG-3’ | 5’-CTGCACGATGAGCTACGGGAGGG-3’ |
| c.932A>G | Exon 8 | 5’-ACCCACATCAGGGCGGGCTGCAT-3’ | 5’-ATGCAGCCCGCCCTGATGTGGGT-3’ |
| c.962A>G | Exon 8 | 5’-GGTACCTGAGGCTGACGCCCAT-3’ | 5’-ATGGGCGTCAGCCTCAGGTACC-3’ |
| c.1129G>A | Exon 9 | 5’-CATGCCCAACAGTAAGGGCAGC-3’ | 5’-GCTGCCCTTACTGTTGGGCATG-3’ |
